# Neurofilament light chain reflects motor impairment in myotonic dystrophy type 1

**DOI:** 10.3389/fneur.2026.1820238

**Published:** 2026-05-26

**Authors:** Chul Hwi Shin, Incheol Seo, Ye-Ri Kim, Jin-Mo Park, Jin-Sung Park

**Affiliations:** 1 Department of Biomedical Science, The Graduate School, Kyungpook National University, Daegu, Republic of Korea; 2 Department of Immunology, School of Medicine, Kyungpook National University, Daegu, Republic of Korea; 3 Department of Neurology, Dongguk University College of Medicine, Dongguk University Gyeongju Hospital, Gyeongju, Republic of Korea; 4 Department of Neurology, School of Medicine, Kyungpook National University, Kyungpook National University Chilgok Hospital, Daegu, Republic of Korea

**Keywords:** biomarkers, motor impairment, muscular impairment rating scale, myotonic dystrophy type 1, neurofilament light chain

## Abstract

**Introduction:**

Myotonic dystrophy type 1 (DM1) is a genetic disorder caused by CTG trinucleotide repeat expansion in the dystrophia myotonica-protein kinase (DMPK) gene and is characterized by progressive muscle weakness with multisystemic involvement, including the central nervous system (CNS). Neurofilament light chain (NfL) has emerged as a biomarker of neuroaxonal damage in various neurological conditions. This study aimed to evaluate plasma NfL as a biomarker of disease severity by examining its association with motor function in ambulatory patients with DM1.

**Methods:**

Thirty-three DM1 patients and 31 controls were enrolled. Plasma NfL levels were measured, and clinical variables including age, sex, age at onset, and disease duration were collected. Motor function was assessed using the Muscular Impairment Rating Scale (MIRS) and the 6-min walk test (6MWT). Associations between NfL levels and clinical measures were analyzed using partial correlation based on Kendall's Tau with adjustment for age.

**Results:**

Age-adjusted analyses demonstrated a significant positive correlation between NfL levels and MIRS scores (τ = 0.387, *p* = 0.002) and a significant negative correlation with 6MWT performance (τ = −0.344, *p* = 0.014). Disease duration was also positively associated with age-adjusted NfL levels (τ = 0.334, *p* = 0.007). Although NfL levels showed a negative correlation with peroneal CMAP amplitudes, this association was not significant after age adjustment.

**Conclusion:**

Plasma NfL levels were associated with disease duration and motor function severity in DM1. These findings suggest that NfL may serve as a biomarker reflecting not only neuroaxonal damage but also motor impairment in DM1. Larger longitudinal studies are warranted to validate these findings.

## 1 Introduction

Myotonic dystrophy type 1 (DM1) is the most common adult-onset inherited muscular dystrophy and follows an autosomal dominant pattern of inheritance. It is caused by a CTG trinucleotide repeat expansion in the dystrophia myotonica-protein kinase (DMPK) gene, which leads to aberrant alternative splicing of multiple downstream targets. The diagnosis of DM1 is confirmed by the genetic identification of 50 or more CTG repeats in the DMPK gene. Repeat sizes of 50–80 indicate a mild clinical phenotype, while large repeat expansions up to 4,000 indicate a serious clinical phenotype (1–3). Clinically, DM1 is characterized by myotonia and progressive distal muscle weakness. However, it is increasingly recognized as a multisystem disorder with central nervous system (CNS) involvement, manifesting as cognitive impairment, excessive daytime sleepiness, and sleep-disordered breathing, including sleep apnea (4, 5).

Neurofilament light chain (NfL), a structural protein of the neuronal cytoskeleton, has emerged as a sensitive but non-specific biomarker for axonal injury in various neurological disorders (6). Recent studies have demonstrated elevated NfL levels in both cerebrospinal fluid and blood across a spectrum of neurodegenerative and neuroinflammatory diseases. In DM1, several recent studies have reported increased blood NfL concentrations, primarily interpreting these elevations as markers of CNS involvement rather than motor function decline (7–10). Given the evidence of CNS involvement in DM1, NfL may serve as a potential biomarker reflecting overall disease severity and neurological burden.

This study aimed to investigate NfL concentrations in patients with DM1 and explore their association with motor function measures, including the Muscular Impairment Rating Scale (MIRS) and the 6-min walk test (6MWT). Considering the known influence of age on NfL levels, we additionally performed analyses within an age-restricted subgroup (52–70 years) to further assess whether NfL reflects motor impairment independent of age-related effects.

## 2 Materials and methods

### 2.1 Participants

We enrolled a cohort of 33 patients with a genetic diagnosis of DM1 defined by the presence of CTG repeat expansion in DMPK gene. In addition, we recruited 31 healthy controls without any known neurological or genetic disease. Clinical data and plasma samples from patients with DM1 were collected during routine outpatient visits between January 2019 and June 2023. All clinical and laboratory variables were assessed at the time of sample collection. Baseline clinical characteristics, laboratory data, motor functional scales, pulmonary function, and electrophysiological data were obtained. Inclusion criteria for DM1 patients were: (1) genetically confirmed DM1 and (2) availability of plasma samples and clinical data. Patients with other neurological disorders or acute systemic illnesses that could influence NfL levels were excluded. Controls were selected based on the absence of neurological or systemic diseases. This study was approved by the Institutional Review Board of Kyungpook National University Chilgok Hospital (KNUCH-2019-06-018).

### 2.2 Acquisition of plasma samples and measurement of NfL levels

All plasma samples were processed within 2 h after collecting 10 ml of blood from the subjects, and ethylenediaminetetraacetic acid tubes were used for blood collection. The plasma was separated by spinning at 3,000 rpm for 15 min and then frozen at −80 °C in sterilized microtubes after temporary storage at −20 °C for up to 48 h.

The NfL level measurements of the plasma samples were performed using an ultrasensitive immunoassay on a Single-Molecule Assay (SiMoA) platform (Bredis Healthcare, Korea). Specifically, the assay was performed using the commercially available SiMoA NF-light V2 Advantage kit (Quanterix, item 104073), run on the fully automated ultrasensitive SiMoA HD-X Analyzer (Quanterix Corporation, Massachusetts), and all samples were measured in duplicate using one batch of reagents from the same lot in a single round of experiments. The NfL levels from duplicate measurements were averaged to obtain the mean concentration, and one outlier was excluded from the analysis.

### 2.3 Diagnostic data for statistical analysis

The clinical data, including sex, age at onset, and disease duration, were retrieved. The serum variables such as creatine kinase, albumin, erythrocyte sedimentation rate, C-reactive protein, myoglobin, creatinine, liver function tests, glucose, and plasma NfL levels were collected. The Muscular Impairment Rating Scale (MIRS) and 6-min walk test (6MWT) results were also collected.

MIRS is a scale of 1 to 5 points, with higher numbers indicating more severe muscle weakness: grade 1 = no muscular impairment, grade 2 = minimal signs such as myotonia or facial weakness, grade 3 = distal weakness, grade 4 = mild to moderate proximal weakness, and grade 5 = severe proximal limb weakness. The 6-min walk test (6MWT) is a cardiopulmonary and motor function test, widely used as an outcome measure in neuromuscular disorders. Patients are instructed to walk for 6 min at their fastest pace, using assistive devices if these are part of their usual mobility. The total distance covered during the 6-min period is recorded.

The pulmonary function test included forced vital capacity (FVC) and forced expiratory volume in 1 second (FEV_1_). The electrophysiology study encompassed conventional nerve conduction studies that included compound motor action potentials of the median and peroneal nerves. The sensory nerve action potentials of the median and superficial peroneal nerves were also included.

### 2.4 Statistical analysis

The NfL levels between the DM1 and healthy control groups were compared using the Mann–Whitney *U*-test. Given the considerable age difference between the groups (mean age of 40.45 for DM1 vs. 67.52 for controls), a subset analysis was conducted, including only individuals aged between 52 and 70 years—the overlapping age range of both groups—followed by a Mann–Whitney *U*-test.

Validation of the observed differences in NfL levels between the DM1 and control groups involved the use of analysis of covariance (ANCOVA) and multivariable linear regression models, with adjustments for age and sex. Additionally, propensity score matching was performed, based on age alone and on both age and sex. After matching, NfL levels of the matched samples were compared using the Mann–Whitney *U*-test.

Linear regression analysis explored the impact of aging on NfL levels in both groups. Associations between NfL concentrations and clinical measures—including motor function scales, nerve conduction study findings, and laboratory parameters—were examined using partial correlation analysis based on Kendall's Tau, while adjusting for age alone or for both age and sex. These analyses were performed using the “ppcor” package in R.

A *p*-value less than 0.05 was considered statistically significant. All statistical analyses and visualizations were conducted using R, with graphical outputs generated via the “ggplot2” package. For selected analyses, age-adjusted NfL values were interpreted in light of published age-dependent reference models for serum NfL (11).

## 3 Results

DM1 patients enrolled in this study had a mean age of 40.45 ± 12.86 years; the age at onset was 25.30 ± 13.52 years, and disease duration was 15.0 ± 7.81 years. The mean MIRS score was 3.13 ± 1.01, and the 6MWT distance was 303.26 ± 144.82 meters. The 31 healthy controls enrolled in the study showed a mean age of 67.52 ± 6.51 years. Baseline characteristics of DM1 patients and controls are summarized in Table 1, highlighting a substantial age difference between groups, which motivated subsequent age-adjusted analyses.

**Table 1:** Clinical characteristics and demographics of the myotonic dystrophy type 1(DM1) patients and controls.

Variable	DM1 group (*n* = 33)	Control (*n* = 31)	*p*-value
Male, *n* (%)	20 (60.61%)	3 (9.68%)	0.009
Age, years (SD)	40.45 (12.86)	67.52 (6.51)	< 0.001
Age-of-onset, years (SD)	25.3 (13.52)		
Disease duration, years (SD)	15 (7.81)		
NfL, pg/ml (SD)	15.57 (8.82)	13.14 (3.79)	0.29
MIRS	3.13 (1.01)		
6MWT, m (SD)	303.26 (144.82)		
FVC, % (SD)	66.57 (20.15)		
FEV1, % (SD)	71.97 (21.4)		
AST, U/L (SD)	45.06 (19.60)		
ALT, U/L (SD)	52.87 (36.19)		
Albumin, g/dl (SD)	4.3 (0.3)		
Creatinine, mg/dl (SD)	0.64 (0.18)		
CRP, mg/dl (SD)	0.6 (1.18)		
CK, U/L (SD)	278.55 (133.55)		
ESR, mm/h (SD)	25.03 (19.5)		
Glucose, mg/dl (SD)	128.66 (89.16)		
Myoglobin, ng/ml (SD)	139.47 (76.26)		
CMAP median, mV (SD)	6.56 (2.28)		
CMAP peroneal, mV (SD)	2.82 (1.94)		
SNAP median, μV (SD)	28.39 (13.12)		
SNAP superficial peroneal, μV (SD)	13.96 (7.78)		

MIRS, muscular impairment rating scale; NfL, neurofilament light chain; 6MWT, 6-min walk test; ALT, alanine aminotransferase; AST, aspartate aminotransferase; CMAP, compound muscle action potential; SNAP, sensory nerve action potential.

**Figure 1:**
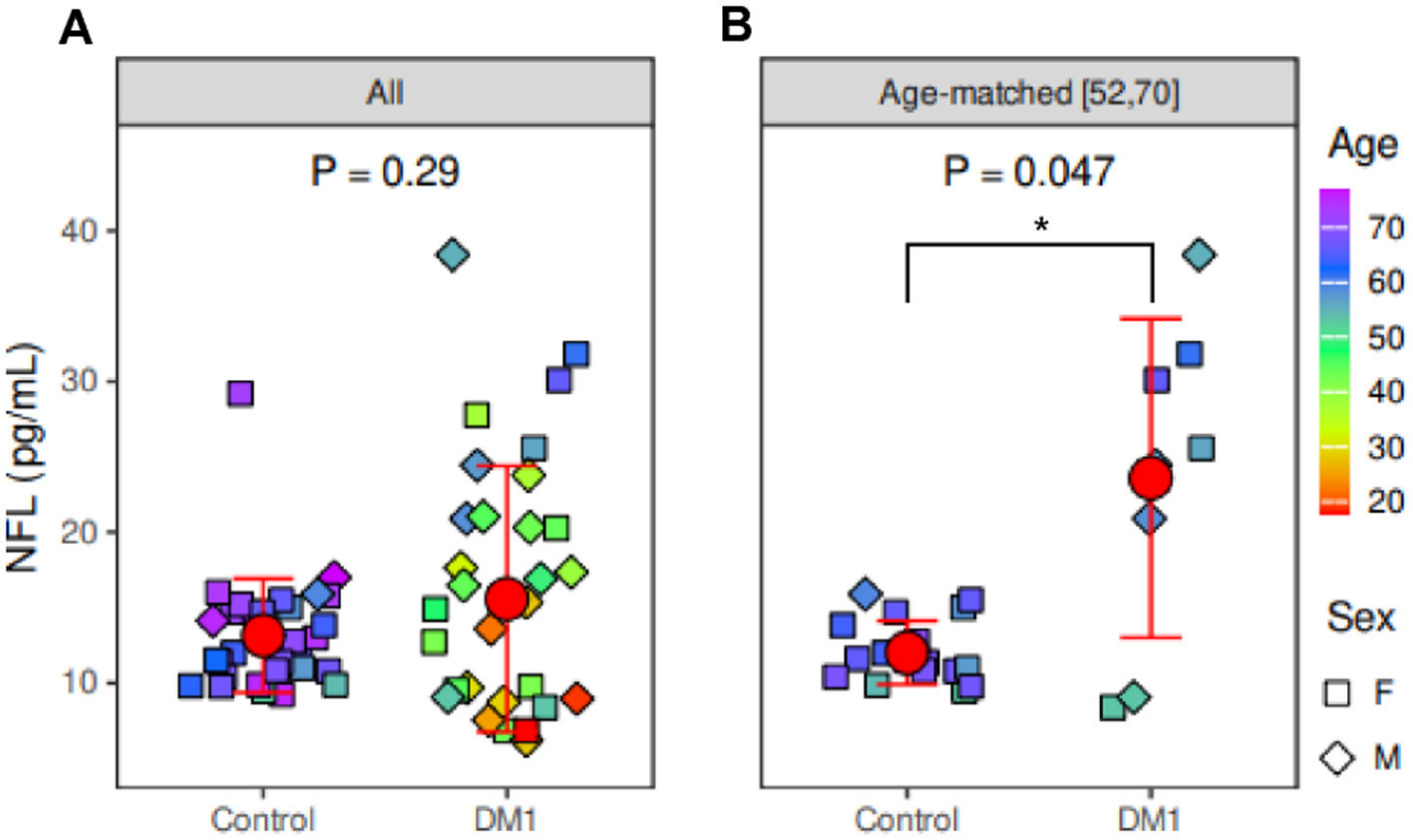
Plasma NfL levels in DM1 patients and controls. **(A)** All participants and **(B)** age-restricted subgroup (52–70 years). Individual data points are shown with group-level central tendency and dispersion. Group comparisons were performed using the Mann–Whitney *U*-test. No significant difference was observed in the full cohort **(A)**, whereas NfL levels were significantly higher in DM1 patients in the age-restricted analysis [**(B)**; *p = 0.047].

In the initial comparison of NfL levels between the DM1 patient group and the healthy control group, no statistically significant difference was observed (Figure 1A). While the control group exhibited relatively low inter-individual variability in NfL levels, the DM1 group showed a broader range of values. Given that the DM1 group had a significantly younger mean age compared to the control group (Table 1), further analyses were conducted to account for the age disparity between the groups. When the age was restricted to 52 years and older (minimum age for the control group) and 70 years and younger (maximum age for the disease group), including 8 patients with DM1, there was a statistically significant difference in NfL levels between the two groups (Figure 1B). These findings indicate that age acts as a major confounder in group-level comparisons of NfL concentrations, and this was further supported by ANCOVA and multivariable linear regression analyses, which adjusted for both age and sex. In addition, propensity score matching based on age alone, as well as on both age and sex, revealed consistent and statistically significant differences in NfL levels between the groups (Table 2).

**Table 2:** Group comparisons of plasma Nfl levels between DM1 patients and controls using age- and sex-adjusted model.

Analysis	Variables adjusted	Statistics	Value	*p*-value
ANCOVA (31 control vs. 33 DM1)	Age, sex	*F*	2.98	0.089
Linear regression (31 control vs. 33 DM1)	Age, sex	Coefficient	12.23	**< 0.001**
Propensity score matching (5 control vs. 3 DM1) + Mann–Whitney *U*-test	Age, sex	*W*	0	0.371
Propensity score matching (7 control vs. 5 DM1) + Mann–Whitney *U*-test	Age	*W*	0	**0.036**

Bold values represent statistically significant results (*p* < 0.05).

**Figure 2:**
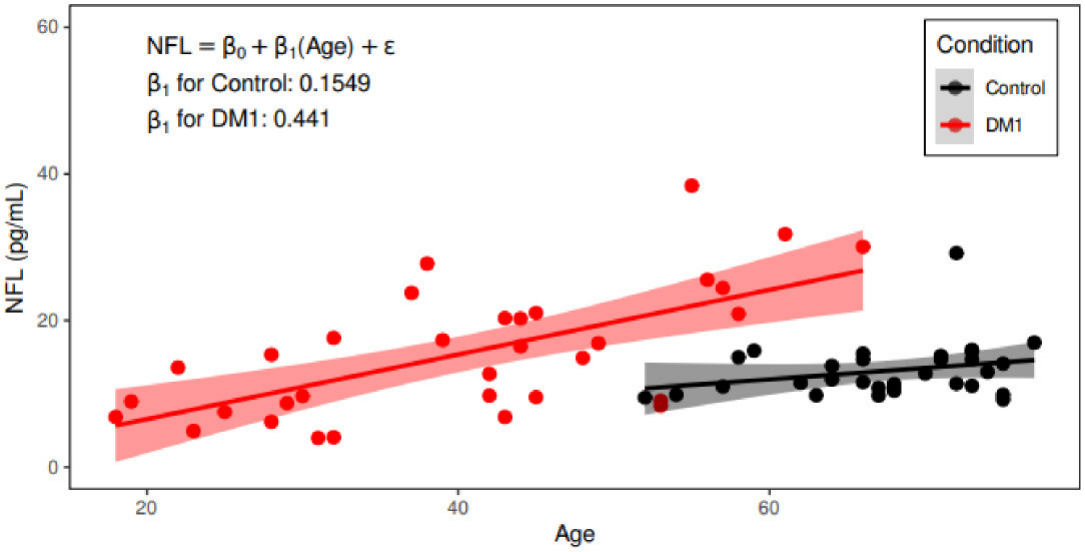
Age-related changes in plasma NfL levels in DM1 patients and controls. Lines represent predicted values from linear regression models of NfL as a function of age, fitted separately for DM1 patients (red) and controls (black). Predictions are shown only within the observed age range. DM1 patients exhibited a steeper age-related increase in NfL levels compared with controls.

Given that NfL levels are known to increase with age (6, 11), we assessed the effect of aging on NfL concentrations in both groups using linear regression analysis. The results showed that the regression coefficient was higher in the DM1 group compared to the healthy control group, indicating a more pronounced age-related increase in NfL levels among individuals with DM1 (Figure 2).

Therefore, we considered the possibility that the well-known age-related increase in NfL could partially mask group differences in mean NfL levels. When analyzing correlations with clinical variables, we therefore used age-adjusted models to account for the age effect on NfL. We also examined laboratory parameters that may affect NfL levels, such as AST, ALT, glucose, and creatinine, and found that they were not significantly associated with NfL levels in patients with DM1 (Supplementary Table 1) (11).

**Figure 3:**
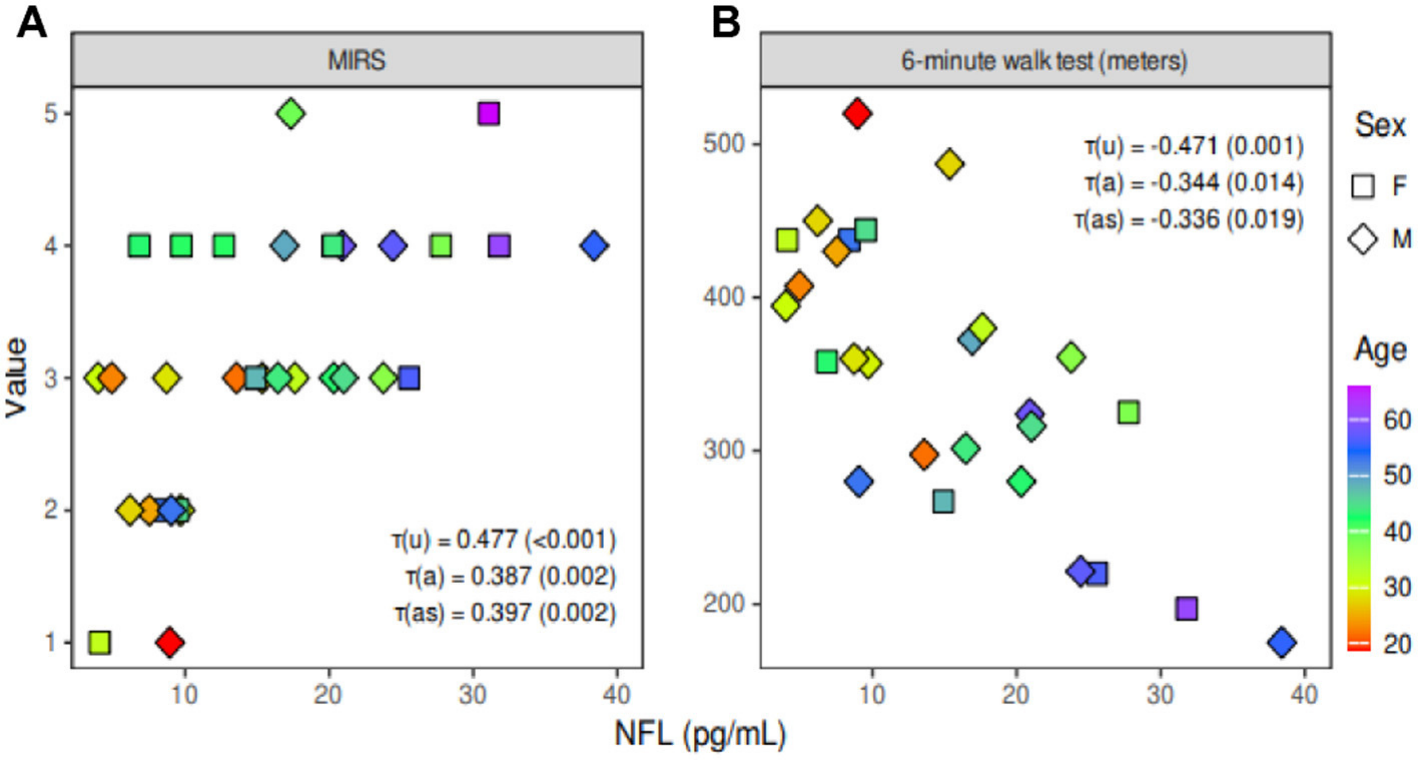
Associations between plasma NfL levels and motor function measures in DM1 patients. **(A)** Correlation between NfL levels and MIRS score (*n* = 32). **(B)** Correlation between NfL levels and 6MWT distance (*n* = 27). Associations were assessed using Kendall's tau correlation, with unadjusted and age- or age- and sex-adjusted coefficients shown in the panels.

To evaluate whether disease progression in DM1 patients is reflected in NfL levels, we explored correlations between the MIRS score, which measures muscle disease severity, the 6MWT results, which evaluate motor function, and NfL levels (Table 3). Before adjustment, NfL levels showed a strong positive association with the MIRS score (τ = 0.477, *p* < 0.001). After adjustment for age and after adjustment for both age and sex, the correlations were somewhat lower but remained statistically significant (τ = 0.387, *p* = 0.002 and τ = 0.397, *p* = 0.002, respectively). Thus, even when accounting for the age-related increase in NfL levels, higher NfL levels in DM1 patients were associated with higher MIRS scores (Figure 3A).

**Table 3:** Correlation between plasma NfL and clinical variables in DM1 patients.

Variable	Unadjusted	Age-adjusted	Age + sex-adjusted
MIRS (*n* = 32)	τ = 0.477, *p* < 0.001	τ = 0.387, *p* = 0.002	τ = 0.397, *p* = 0.002
6MWT (*n* = 27)	τ = −0.471, *p* < 0.001	τ = −0.344, *p* = 0.014	τ = −0.397, *p* = 0.019
Disease duration (*n* = 33)	τ = 0.383, *p* = 0.002	τ = 0.334, *p* = 0.007	τ = 0.334, *p* = 0.008

For the 6MWT, an inverse relationship with NfL was observed. In unadjusted analyses, higher NfL levels were associated with shorter 6MWT distances (τ = −0.471, *p* < 0.001). After adjustment for age and for both age and sex, the correlations were attenuated but remained statistically significant (τ = −0.344, *p* = 0.014 and τ = −0.397, *p* = 0.019, respectively), indicating that higher NfL levels are associated with worse ambulatory performance even after accounting for age (Figure 3B).

**Figure 4:**
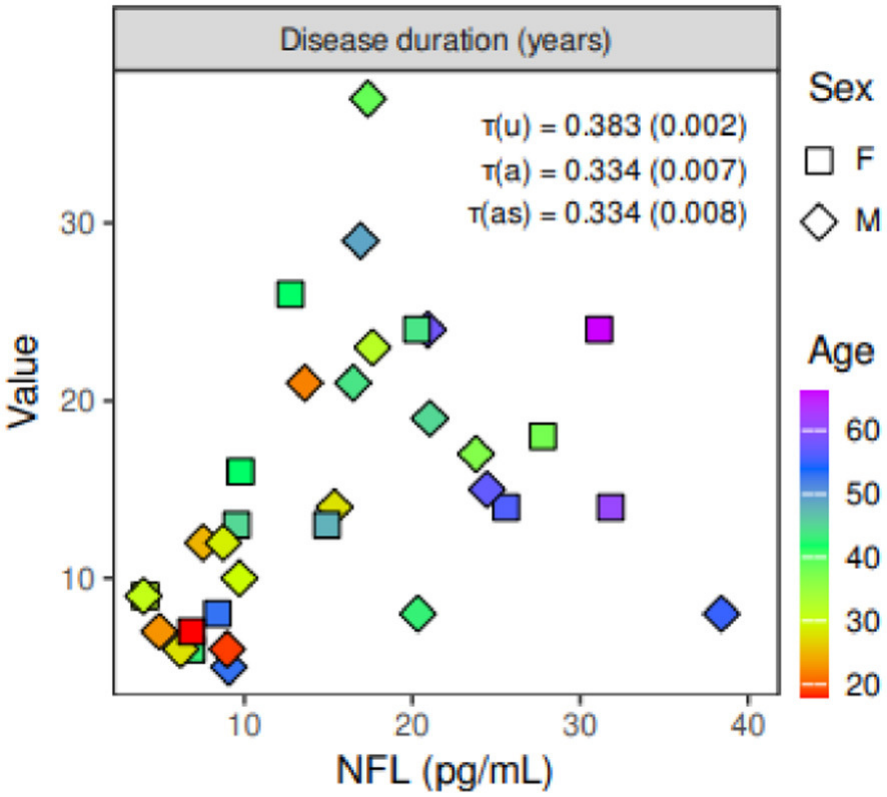
Association between plasma NfL levels and disease duration in DM1 patients (*n* = 33). Correlations were assessed using Kendall's tau, with unadjusted and age- or age- and sex-adjusted coefficients shown.

Longer disease duration, the clinical variable most strongly linked to age, was associated with higher NfL levels in unadjusted analysis (τ = 0.383, *p* = 0.002), and this association remained significant in models adjusted for age or for both age and sex (τ = 0.334, *p* = 0.007 and τ = 0.334, *p* = 0.008, respectively; Figure 4). By contrast, age at onset of DM1 did not show a significant association with NfL levels after age adjustment, suggesting that NfL is more closely related to accumulated disease burden than to the timing of symptom onset.

To further explore neurophysiological correlates, we evaluated NfL levels in relation to NCS parameters. In unadjusted analysis, we observed a negative correlation between NfL levels and peroneal CMAP amplitudes (τ = −0.310, *p* = 0.024), but the statistical significance could not be confirmed after adjusting for age or for both age and sex (τ = −0.236, *p* = 0.090 and τ = −0.239, *p* = 0.094, respectively). The median CMAP showed no correlation with NfL levels (τ = −0.213, *p* = 0.114). These trends are consistent with distal motor involvement in DM1 but suggest that the current sample size is insufficient to conclusively link peripheral electrophysiological measures to NfL in age-adjusted models.

## 4 Discussion

In this study, we demonstrated that plasma NfL levels are elevated in patients with DM1 compared with controls after appropriate adjustment for age and sex, and that NfL is moderately but consistently associated with motor impairment as measured by both MIRS and 6MWT. Furthermore, disease duration—but not age at onset—was positively associated with NfL after adjustment for age, suggesting that NfL reflects cumulative disease burden rather than merely chronological aging. Taken together, these findings support plasma NfL as a candidate biomarker of disease severity in DM1, with particular relevance to motor function in ambulatory patients.

Several recent studies have systematically examined NfL in DM1. Nicoletti et al. (7) reported elevated serum NfL levels in DM1 compared with controls, with correlations to age and selected cognitive measures, and interpreted NfL primarily as an indicator of CNS damage in DM1. van der Plas et al. (8) similarly showed that blood-based markers of neuronal injury, including NfL, are increased in adult-onset DM1 and associate with white-matter microstructural abnormalities on diffusion imaging, as well as with neurocognitive outcomes. More recently, Takahashi et al. (9) confirmed elevated plasma NfL and phosphorylated tau in a Japanese DM1 cohort, suggesting that these markers may capture DM1-related neurodegenerative processes and could be useful in future biomarker-guided therapeutic monitoring. A comprehensive review by Rossi and Silvestri (10) synthesized these and other fluid biomarker data, concluding that CSF and blood NfL are currently the most robust biomarkers of CNS involvement in DM1. Our findings are broadly consistent with this literature in demonstrating elevated NfL in DM1 and a clear age-dependence of NfL levels. However, in contrast to prior studies that have primarily focused on CNS-related outcomes, our study specifically examined the relationship between NfL and motor function. Nicoletti et al. (7) did not detect a significant association between NfL and MIRS, likely reflecting the relatively mild motor impairment in their cohort (mean MIRS of approximately 2.4). Similarly, van der Plas et al. (8) emphasized relationships between NfL and white-matter changes, as well as cognitive and behavioral outcomes, while motor function was captured more indirectly through imaging-derived correlates. In contrast, our cohort consisted of patients with more advanced muscle involvement (mean MIRS of approximately 3.1, with a substantial proportion in grades 3–5), which may have increased the dynamic range for detecting associations with motor scales. Importantly, our study is, to our knowledge, among the first to demonstrate significant associations between plasma NfL levels and both a clinician-rated motor severity scale (MIRS) and a performance-based ambulatory measure (6MWT) in DM1. The convergent associations with these complementary motor endpoints suggest that NfL may capture aspects of the multisystem pathology in DM1 that extend beyond purely CNS-mediated cognitive or behavioral manifestations. Given that respiratory and limb muscle weakness critically determine morbidity and mortality in DM1 (12–14), a biomarker that correlates with motor function has direct clinical relevance.

A large body of neuroimaging work has established DM1 as a disorder with widespread CNS involvement, particularly affecting white matter. Early voxel-based morphometry and volumetric MRI studies reported cerebral atrophy and diffuse white-matter abnormalities in DM1 (15–18). Diffusion tensor imaging and tractography have consistently shown reduced fractional anisotropy in major white-matter tracts, including the corticospinal tract (CST), with associations to both motor and cognitive deficits (5, 19–22). Longitudinal MRI data suggest that these abnormalities progress over time, but the rate of change can be slow and heterogeneous, making MRI less practical as a standalone biomarker in routine clinical practice or in short-to-medium-term trials (18, 23). Histopathological studies further support the presence of CNS degeneration in DM1, including tau pathology and white-matter changes (24, 25). Fluid biomarker studies in CSF have shown alterations in tau and amyloid-β in a subset of DM1 patients, although results have been more variable than for NfL (26, 27). Recent work combining tau PET, CSF, and plasma biomarkers underscores the complexity of DM1-related neurodegeneration and suggests that multiple pathological pathways may coexist (28). Within this context, our findings complement the existing imaging and pathology literature. Prior work from our group showed that CST microstructural abnormalities on diffusion imaging are associated with motor dysfunction in adult-onset DM1 (5). The present study extends these observations by demonstrating that plasma NfL—which is easier to measure repeatedly than MRI—also correlates with motor impairment. When taken together with reports linking NfL to white-matter abnormalities and cognitive dysfunction (7, 8, 10), our data support a model in which NfL reflects a combination of CNS involvement and global neuroaxonal damage that ultimately manifests as both cognitive and motor decline.

An interesting aspect of our results is the dissociation between disease duration and age at onset. Disease duration showed a robust positive association with NfL levels, even after age adjustment, whereas age at onset did not. This pattern suggests that NfL primarily indexes the accumulated burden of disease rather than the timing of clinical onset. Given the well-known difficulties in precisely determining onset in DM1—where symptoms may evolve insidiously over years—duration is itself an imperfect measure. Nevertheless, the persistence of the association after controlling for age supports the idea that NfL is sensitive to cumulative disease activity rather than merely reflecting physiological aging. These findings echo the broader DM1 literature in which disease duration, CTG repeat length, and CNS imaging abnormalities have all been linked to clinical severity, but not always in a simple linear manner (16, 18, 20–22). Our data add to this by suggesting that NfL could serve as an integrative marker capturing the combined impact of aging and disease progression, especially in patients with relatively early onset who have lived with the disease for many years.

Although NfL has traditionally been viewed as a marker of CNS axonal injury, it is also expressed in peripheral nerves and, to a lesser extent, in muscle. Studies in other myopathies and neuropathies have shown that NfL can be elevated in conditions with prominent peripheral nervous system (PNS) involvement (29). In our cohort, peroneal CMAP amplitudes showed a trend toward negative correlation with NfL that did not remain significant after age adjustment. Given the small sample size and the distal-predominant weakness characteristic of DM1, this pattern is not unexpected. It suggests that PNS may contribute to circulating NfL, but our study was underpowered to disentangle CNS and PNS sources. Thus, the contribution of peripheral vs. central pathology to circulating NfL in DM1 remains to be clarified. Future multimodal studies combining NfL with detailed NCS/EMG, muscle imaging, and CNS MRI will be needed to clarify the relative contributions of central vs. peripheral pathology to NfL elevations in DM1. Nevertheless, from a clinical standpoint, the key point is that higher NfL levels were associated with worse motor function.

From a clinical perspective, our findings support plasma NfL as a minimally invasive, quantitative marker that correlates with motor impairment in DM1. Because blood sampling is relatively inexpensive and can be repeated frequently, NfL is well suited for use as a monitoring tool in both routine practice and clinical trials. In particular, its association with 6MWT—a widely used endpoint in neuromuscular trials—suggests that NfL might serve as an adjunctive biomarker to capture subclinical changes that precede measurable declines in walking distance. For future interventional studies, NfL could potentially be used as a pharmacodynamic marker to quantify treatment effects on neuroaxonal integrity. However, the moderate effect sizes observed in our cohort and the strong influence of age highlight the need for careful adjustment for demographic factors and for within-subject longitudinal designs. Incorporating age-adjusted reference models or z-scores for NfL (11) may improve comparability across cohorts and time points.

Several limitations of our study warrant consideration. First, the sample size was modest, limiting statistical power—particularly for age-adjusted analyses involving electrophysiological parameters and subgroup analyses. Second, the cross-sectional design precludes any conclusions about the temporal relationship between changes in NfL and clinical progression. Longitudinal studies will be essential to determine whether intra-individual NfL trajectories track motor decline or response to treatment. Third, because our control group was substantially older than the DM1 cohort, we conducted a targeted sub-analysis by restricting the age range to 52–70 years. This specific range represents between the minimum age of our controls and the maximum age of our disease group, allowing for a more direct comparison while minimizing age-related physiological NfL elevations. This subgroup analysis included 8 DM1 patients and should be interpreted with caution due to the limited sample size. Although the age-restricted subgroup included a relatively small number of patients, the consistency of findings across multiple analytical approaches—including age-adjusted models, regression analyses, and propensity score matching—supports the robustness of our results. Fourth, our study included only ambulatory patients, and the 6MWT cannot be applied to non-ambulatory individuals. As a result, the generalizability of our findings to more advanced DM1 stages is limited. Future work should evaluate NfL in relation to alternative motor scales that capture non-ambulatory patients and to respiratory outcomes, which are highly relevant for prognosis. Finally, we did not systematically assess cognitive or behavioral features in this cohort, precluding a direct comparison of NfL correlations with motor vs. cognitive domains. Despite these limitations, our study adds an important dimension to the emerging DM1 NfL literature by demonstrating clear associations between NfL and clinically meaningful motor outcomes in a cohort with moderate to severe muscle involvement.

## 5 Conclusion

Our findings support plasma NfL as a promising biomarker in DM1 that complements existing CNS-focused imaging and fluid markers. By showing that NfL correlates with both clinician-rated (MIRS) and performance-based (6MWT) motor measures, we provide evidence that NfL reflects not only CNS pathology but also the broader neuromuscular disease burden. Larger, longitudinal, and multimodal studies are needed to confirm these observations, to clarify the relative contributions of central and peripheral pathology, and to establish NfL as a surrogate endpoint for future DM1 clinical trials.

## Data Availability

The data supporting the findings of this study are available from the corresponding author upon reasonable request.
