# Supplementary material for: Neurofilament light chain reflects motor impairment in myotonic dystrophy type 1
Source: Front Neurol. 2026 May 26;17:1820238. doi: 10.3389/fneur.2026.1820238 (PMC13247362; doi:10.3389/fneur.2026.1820238)
Supplement: Supplementary file 1 [file Table_1.docx]

Supplementary Table 1. Correlations between plasma NfL levels and clinical features in DM1 patients using unadjusted, age- and sex-adjusted models.

| **Variable** | **Unadjusted (τ, p)** | **Age-adjusted (τ, p)** | **Age + Sex-adjusted (τ, p)** |
| --- | --- | --- | --- |
| MIRS | 0.477, <0.001 | 0.387, 0.002 | 0.397, 0.002 |
| Disease duration | 0.383, 0.002 | 0.334, 0.007 | 0.334, 0.008 |
| 6MWT | -0.471, <0.001 | -0.344, 0.014 | -0.336, 0.019 |
| FVC | -0.219, 0.090 | -0.178, 0.174 | -0.166, 0.216 |
| FEV1 | -0.201, 0.120 | -0.143, 0.275 | -0.133, 0.321 |
| CK | -0.078, 0.541 | -0.062, 0.632 | -0.069, 0.597 |
| Myoglobin | 0.360, 0.010 | 0.262, 0.067 | 0.259, 0.076 |
| Albumin | -0.011, 0.939 | 0.027, 0.838 | 0.035, 0.799 |
| Glucose | 0.129, 0.329 | 0.084, 0.533 | 0.084, 0.541 |
| AST | 0.004, 0.973 | -0.038, 0.765 | -0.041, 0.754 |
| ALT | -0.004, 0.973 | -0.038, 0.767 | -0.045, 0.729 |
| ESR | 0.219, 0.098 | 0.109, 0.417 | 0.119, 0.384 |
| CRP | 0.002, 0.985 | -0.026, 0.844 | -0.052, 0.705 |
| Creatinine | -0.037, 0.772 | 0.034, 0.789 | 0.007, 0.956 |
| CMAP median | -0.213, 0.114 | -0.257, 0.060 | -0.256, 0.067 |
| SNAP median | 0.029, 0.828 | 0.057, 0.675 | 0.058, 0.679 |
| CMAP peroneal | -0.310, 0.024 | -0.236, 0.090 | -0.239, 0.094 |
| SNAP superficial peroneal | -0.251, 0.067 | -0.146, 0.296 | -0.145, 0.309 |

*τ = Kendall's tau* correlation coefficient
